# Immune Mechanisms of Resistance to Cediranib in Ovarian Cancer

**DOI:** 10.1158/1535-7163.MCT-21-0689

**Published:** 2022-03-21

**Authors:** Ganga Gopinathan, Chiara Berlato, Anissa Lakhani, Ludmila Szabova, Colin Pegrum, Ana-Rita Pedrosa, Florian Laforets, Eleni Maniati, Frances R. Balkwill

**Affiliations:** 1 Barts Cancer Institute, Barts and The London School of Medicine and Dentistry, Queen Mary University of London; London, United Kingdom.; 2 Frederick National Laboratory for Cancer Research, Tumour Microenvironment Leidos Biomedical Research Inc, Frederick, Maryland.

## Abstract

This article investigates mechanisms of resistance to the VEGF receptor inhibitor cediranib in high-grade serous ovarian cancer (HGSOC), and defines rational combination therapies. We used three different syngeneic orthotopic mouse HGSOC models that replicated the human tumor microenvironment (TME). After 4 to 5 weeks treatment of established tumors, cediranib had antitumor activity with increased tumor T-cell infiltrates and alterations in myeloid cells. However, continued cediranib treatment did not change overall survival or the immune microenvironment in two of the three models. Moreover, treated mice developed additional peritoneal metastases not seen in controls. Cediranib-resistant tumors had intrinsically high levels of IL6 and JAK/STAT signaling and treatment increased endothelial STAT3 activation. Combination of cediranib with a murine anti-IL6 antibody was superior to monotherapy, increasing mouse survival, reducing blood vessel density, and pSTAT3, with increased T-cell infiltrates in both models. In a third HGSOC model, that had lower inherent IL6 JAK/STAT3 signaling in the TME but high programmed cell death protein 1 (PD-1) signaling, long-term cediranib treatment significantly increased overall survival. When the mice eventually relapsed, pSTAT3 was still reduced in the tumors but there were high levels of immune cell PD-1 and Programmed death-ligand 1. Combining cediranib with an anti–PD-1 antibody was superior to monotherapy in this model, increasing T cells and decreasing blood vessel densities. Bioinformatics analysis of two human HGSOC transcriptional datasets revealed distinct clusters of tumors with IL6 and PD-1 pathway expression patterns that replicated the mouse tumors. Combination of anti-IL6 or anti–PD-1 in these patients may increase activity of VEGFR inhibitors and prolong disease-free survival.

## Introduction

Over the last decade there has been some improvement in the treatment of high-grade serous ovarian cancer (HGSOC), with targeted therapies such as antiangiogenic agents (1) but many patients develop resistance to these agents (2). The VEGFR inhibitor, cediranib (3) improves progression-free and overall survival when combined with chemotherapy and PARP inhibitors (4) but little is known about mechanisms of resistance to this agent. A small phase I study investigated a combination of cediranib, olaparib, and durvalumab [anti–programmed death-ligand 1 (PD-L1) antibody] in gynecologic cancers (5). Although there were some favorable outcomes, patient sample size was limited. Nevertheless, such trials highlighted the potential for combining angiogenesis inhibitors with other biological and immunotherapy agents in HGSOC.

We have previously investigated the role of IL6 in regulating the inflammatory cytokine network found in HGSOC (6, 7) and in a series of *ex vivo* and human tumor xenograft experiments, found that this cytokine directly stimulated angiogenesis with defective pericyte coverage (8). These studies on IL6-related inflammation and cancer has led to some promising preclinical data on inhibitors of IL6 and related signaling pathways (9). However, this has not translated into therapeutic benefit in early-phase clinical trials of anti-IL6 or anti-IL6 receptor antibodies, although durable responses were seen in Castelman disease, a rare IL6-driven lymphoproliferative disease (10). In a small phase II study of the anti-IL6 antibody siltuximab in advanced HGSOC, we reported periods of disease stabilization and reductions in circulating CCL2, CXCL12, VEGF, and C-reactive protein in the treated patients, but no durable responses (7). However, platelet counts were reduced providing evidence both for biological activity and involvement of IL6 in the paraneoplastic thrombocytosis often associated with HGSOC (11). Other immunotherapies have failed to make an impact in HGSOC with little evidence of activity of immune checkpoint blockade (12) although combination with PARP inhibitors is showing some promise (13) and many other combination trials are underway.

HGSOC has a complex tumor microenvironment (14–16) but our understanding of the effects of targeted and immune therapies, and preclinical study of their combination, has been hindered by the lack of translationally relevant mouse models. We recently described new orthotopic syngeneic HGSOC models with relevant genetic mutations (17). The transcriptional profile, immune, vasculature, and extracellular matrix (ECM) characteristics of these models showed significant similarities with their human counterparts.

We have used these HGSOC models to explore mechanisms of action and potential modes of resistance to cediranib, with the aim of finding preclinical evidence for more effective combination therapies. Long-term cediranib treatment revealed different modes of resistance that were mediated by activation of the IL6/JAK-STAT or programmed cell death protein 1 (PD-1) signaling pathways. Treatment of established peritoneal disease with combinations of cediranib and anti-IL6 or anti–PD-1 antibodies was superior to the monotherapies, resulting in prolonged mouse survival. Using publicly available human datasets, we identified similar subgroups associated with high IL6, JAK-STAT, PD-1, and angiogenesis signatures that again correlated with the distinct TME characteristics observed in our mouse models. Our data indicate that combination of antiangiogenic agents with anti-IL6 or anti–PD-1 therapy may be effective in subgroups of patients.

## Materials and Methods

### Tumor cell lines

HGSOC mouse orthotopic cell lines; 30200 and 60577 were derived from serous ovarian cancer genetically engineered mouse models (GEMM; ref. 15) and engineered to express *Trp53*^−/−^, *Brca1*^−/−^, and *Rb* inactivation. Following intraperitoneal cell injection, they form extensive disease in the omentum and also metastasize to the spleenoportal fat, lesser omentum, and mesentery. The two models vary in their average survival time with 20 weeks (30200) and 6 weeks (60577). HGS2 cell line was derived from a *Pax-8*-cre inducible GEMM driving inactivation of *Trp53, Brca2*, and *Pten* in the fallopian tube epithelium. This model is syngeneic with C57 BL/6J background and upon intraperitoneal cell injection form extensive disease in the omentum and peritoneum similar to the other models by 12 to 14 weeks.

These orthotopic transplantable lines were grown in DMEM/F12 Ham medium (Sigma-Aldrich) constituted with 2% FBS (Gibco-Invitrogen), 100 units/mL penicillin G sodium, and 100 µg/mL streptomycin sulfate (Invitrogen). The medium was also supplemented with 5 mL of 100× Insulin/Transferrin/Selenium (Invitrogen), 5 mL of 50 µg/mL of Hydrocortisone (Sigma-Aldrich), 5 mL of 100x anti-anti (Gibco), and 500 µl of 10 µg/mL murine EGF. Cells were trypsinized with 0.25% trypsin-EDTA (Sigma-Aldrich) and split 1:4.

### Mouse lung endothelial cell line

Mouse lung endothelial cell line (MLEC) was kindly given by Professor Kairbaan Hodivala-Dilke, was used for *in vitro* studies. This cell line was isolated and cultured as described previously (36). MLEC were grown and cultured in endothelial growth medium (HPA laboratories) and maintained within 3 to 4 passages.

### *In vivo* studies

All animal experiments have been conducted in accordance with Animals (Scientific Procedures) Act 1986, and under the license PBE3719B3 with the approval of Queen Mary University of London (QMUL; London, United Kingdom) Ethics committee, an Institutional Animal Care and Use Committee (IACUC).

Procedures observed the guidelines approved by the ethics committees of QMUL under the Home Office Project license PBE3719B3. For survival experiments, mice were culled when they reached humane endpoint as defined in the license.

**Figure 1.**
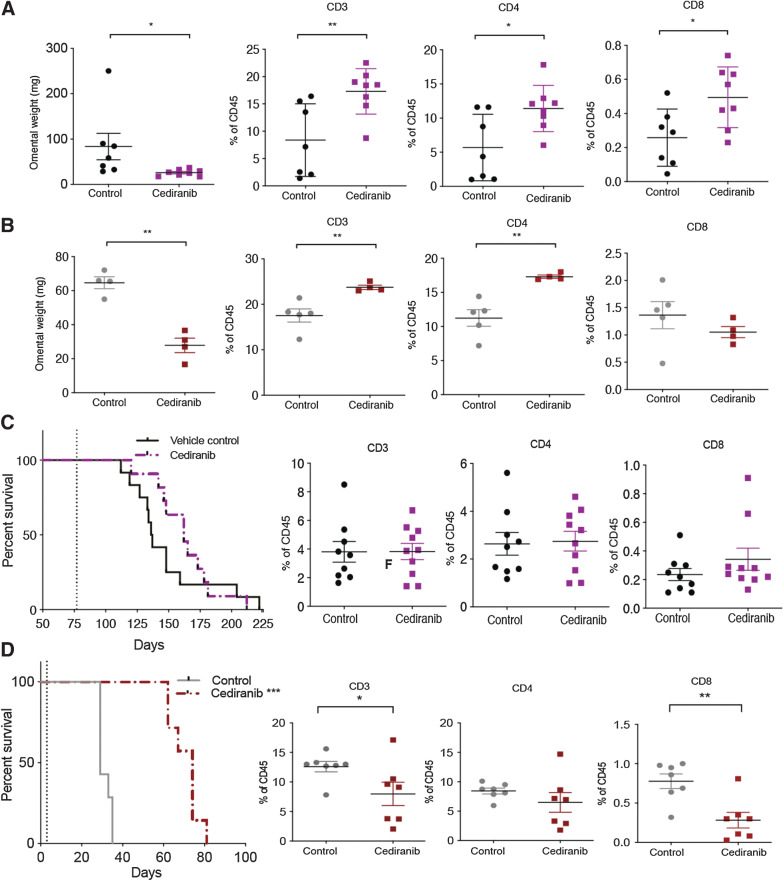
Short-term and survival effects of cediranib treatment on immune microenvironment in two HGSOC mouse models. Mice injected with 30200 or 60577 were given oral gavage; vehicle control or cediranib 5 mg/kg five times a week starting at 10 weeks (30200) or 3 days (60577) after cell injection for a period of 4 to 5 weeks (short-term treatment) or until endpoint (survival indicated by dotted lines). **A,** Omental weight and flow cytometric analysis of lymphoid infiltrate in 30200 short-term treated tumors (*n* = 4). **B,** Omental weight and flow cytometric analysis of lymphoid infiltrate in 60577 short-term treated tumors (*n* = 7). **C,** Survival curve and flow cytometric analysis of lymphoid infiltrate in 30200 model (*n* = 9–10). **D,** Survival curve and flow cytometric analysis of lymphoid infiltrate in 60577 model (*n* = 7).

1 × 10^7^ cells diluted in 300 µL were injected i.p. into 8-week-old either FVB/NCrl (60577, 30200) or C57 BL/6J female mice (HGS2 model) from Charles River, UK. Mice were treated with 5 mg/kg cediranib (Selleck Chemicals) or vehicle control (4% DMSO in water) oral gavage daily five times a week starting at 3 days and a week after in 60577, at 10 weeks in 30200, and at 7 weeks in HGS2 model until experimental endpoint. 2 mg/kg anti-IL6 or isotype control (BioXCell) were given twice weekly i.p., starting at 10 weeks in 30200 model or 7 weeks in HGS2 model after cell injection. Anti–PD-1 10 mg/kg or isotype control (Biolegend) was administered i.p. twice a week for 6 weeks starting at week 2 in 60577 model. For short-term experiments (Fig. 1), the mice were treated for a period of 4 to 5 weeks, for survival experiments the treatments continued till endpoint or for 6 weeks in the case of anti–PD-1. Anti–PD-1 treatments were discontinued after 6 weeks due to toxicity related weight loss in mice. For survival experiments, we used 7 to 8 mice per group as described in the figure legends and carried out repeat experiments either in the same model or in different models.

The endpoint of these mice are assessed by a combination of factors like; extensive disease in the peritoneum, abdominal swelling as a result of built up of ascites and other humane endpoint signs including slower movement, hunched posture, labored breathing, and in occasional cases significant weight loss. For all *in vivo* experiments, mice were randomly assigned to treatment groups. In cases where experiments were not blinded, endpoint decisions were made by an independent member of the animal facility.

### Protein extraction from mouse tumors

Using gentle MACS dissociater,75 mg of *in vivo*–treated 30200 and HGS2 tumors were homogenized and lysed in 1 mL of cold RIPA lysis buffer and extraction buffer (Thermo Fisher Scientific) containing Pierce Protease and Phosphatase Inhibitor Mini Tablet (Thermo Fisher Scientific). The lysates were then centrifuged at 1,500 rpm for 10 minutes and supernatants were collected. Samples were always kept on ice between each step. Then, using a probe sonicator set at 40% amplitude, the supernatants were sonicated for 10 to 20 seconds bursts. Sonicated samples were then left on a roller for 30 minutes at 4**°**C. The samples were then centrifuged for 15 minutes at 13,500 rpm at 4°C. Pellets were then discarded and the samples were stored at −20°C.

### Protein extraction from MLEC

2 × 10^5^ MLEC cells were plated in a six-well plate with 2 mL endothelial medium. After the cells were attached, supernatant was removed and cells were treated with varying concentrations of either mouse IL6 (mIL6), VEGF, cediranib, or their combinations in 2 mL of serum-free endothelial cell media for 24 hours. Cells were then washed with PBS and harvested using RIPA buffer (R0278, Sigma-Aldrich) with proteinase and phosphatase inhibitors.

### Western blotting

Western blotting was performed using the Invitrogen NuPAGE System. Protein concentration was calculated using the bicinchoninic acid (BCA) assay according to the manufacturer's protocol. Samples were prepared by adding 30 µg of protein to sample buffer and reducing agent. Proteins were separated in NuPAGE 4% to 12% Bis-Tris gels. Resolved proteins were transferred using Invitrolon PVDF/Filter Paper Sandwiches (Invitrogen) and XCell II Blot Module system (Invitrogen). Immunodetection was performed by adding a substrate for the HRP (Amersham ECL Western Blotting Detection Reagents) followed by an exposure onto the ChemiDoc system. The following antibodies were used phospho-STAT3 (Tyr705), STAT3, phospho-ERK1/2 (Thr202/Tyr204), and ERK1/2 all from Cell Signaling Technology. α-Tubulin was purchased from Sigma-Aldrich. Densitometry analysis normalizing phospho protein against total protein for western blot was carried out using ImageJ software.

### Flow cytometry

Mouse omental tumors were collected in ice-cold PBS and were minced in collagenase from Clostridium histolyticum (2 mg/mL, Sigma-Aldrich), and DNase I from bovine pancreas (25 mg/mL, Sigma-Aldrich) in HBSS (Sigma 1X). The minced tumors were then digested for 20 minutes at 37°C in a shaker. The tissue was then passed through a 70-µm cell strainer and resuspended in flow cytometry buffer (PBS, 2.5% BSA, 2 mmol/L EDTA) and cells counted. Cells were plated in a 96-well plate (2 million cells/well) and resuspended in Fc block CD16/32 (eBioscience,101320) for 15 minutes. Staining antibodies were diluted 1:200 unless differently specified: anti-CD45-BV785 1:100 (Biolegend,103149), anti-CD3 PE-Cy7 1:50 (Biolegend,100320), anti-CD4 BV605 1:100 (Biolegend,100548), anti-CD8 BV710 (eBioscience,17–0081–83), anti-CD11b BV650 (Biolegend,101239), anti-F4/80 PE (Biolegend,123110), anti-CD19 PE Dazzle (Biolegend,115534), anti–PD-1 e450 (eBioscience, 48–9981–82), anti–PD-L1 (BV421, Biolegend,374508), anti-MR FITC (Biolegend,141704), and anti-MHCII APC-Cy7 (Biolegend,107628). Viability was assessed with Fixable Viability Dye eFluor506 (eBioscience, v65–0866–18) diluted 1:200. Staining was performed for 30 minutes at 4°C. The cells were then washed and fixed (1:1 FACS buffer and 4% formalin). Flow cytometric analysis was performed using an LSRFortessa cell analyzer (BD Biosciences) and FACSDiva software Version 10. Data were transferred and analyzed using the FlowJo software (Tree Star).

### IHC

Omental tissues fixed in 4% formaldehyde were transferred to 70% ethanol, paraffin-embedded, and 4-mm sections were cut. These cut sections were used to perform IHC staining.

The slides were first deparaffined by submerging twice in xylene for 5 minutes. Slides were then rehydrated for 2 minutes in each of the following ethanol solutions: 100%, 90%, 70%, 50%, and finally in ddH2O for 3 minutes. Antigen retrieval was performed using the citrate buffer for 30 minutes at 98°C. Following antigen retrieval the slides were washed and treated with 3% H2O2 (Thermo Fisher Scientific, H/1800/15) in PBS for 30 minutes. After peroxide blocking the slides were washed again and blocked with goat or rabbit serum for 45 minutes. The primary antibody was diluted 1:100 for Endomucin (Santa Cruz, Sc-65495), 1:40 for pSTAT3 (Cell Signaling Technology, 9145S), 1:500 Cytokeratin 8 (Abcam, ab53280), 1:100 Ki67 (Abcam, ab16667) in blocking buffer and incubated overnight at 4°C. The following day the slides were washed three times and the secondary antibody i.e., anti-rabbit HRP or anti-rat HRP were added for 45 minutes at room temperature. Color was developed with Diaminobenzidine substrate-chromogen (Dako Liquid DAB+ Substrate Chromogen System, K3468 Dako) and tissues were counterstained with Gill's hematoxylin I (Sigma-Aldrich, GHS1128), washed, dehydrated in ethanol, and mounted in DPX (Sigma-Aldrich, 06522). Additional chromogens, Vector VIP (purple) and AP blue were also used for multiplex staining.

### Immunofluorescence

The rat aortic rings were cultured in a 48-well plate for 7 to 10 days using recombinant rat IL6, VEGF, cediranib, and its combinations. The rings were then washed with PBS, fixed in 4% formaldehyde for 20 minutes. The wells were then washed once in PBS and the rings were permeabilized with 0.5% Triton X-100 in PBS for 30 minutes. Then the wells were washed twice in PBS and stained with 100 μL of BS-1 Lectin FITC (1 mg/mL; Sigma-Aldrich, catalog no. L9381/L5264; 1:200), anti-actin, alpha smooth muscle actin (α-SMA) Cy3 (Sigma-Aldrich, catalog no. C6198; 1:500) overnight at 4°C. The following day, plates were washed twice in PBS and the rings were removed from the 48-well plate, using a syringe needle, placed on a microscope slide and mounted with Prolong Gold DAPI containing medium (Invitrogen, catalog no. P36931). The slides were left to dry and imaged using confocal microscopy (Zeiss LSM 510 META).

### ELISA

Mouse IL6 cytokine concentrations in MLEC treated with recombinant mIL6 and cediranib were measured using Quantikine ELISA kit (R&D Systems) according to the manufacturer's protocol. Absorbance was measured at 450 nm using an Opsys MR plate reader (Dynex Technologies).

### RNAscope

*In-situ* hybridization was completed using the manufacturers’ protocol for RNAscope [Advanced Cell Diagnostics Bio-Techne (ACD)]. Formalin-fixed, paraffin-embedded (FFPE) human HGSOC samples were deparaffinized by being heated for 1 hour at 60˚C and then submerged in xylene twice for 5 minutes. Slides were then submerged in 100% ethanol twice for 1 minute. Tissues were outlined with a hydrophobic barrier pen and treated with company provided hydrogen peroxide for 10 minutes, then rinsed twice in distilled water. The slides were then boiled in target retrieval reagent for 15 minutes, washed in distilled water, dipped in 100% ethanol, and allowed to dry. Tissue sections were permeabilized by incubating with protease plus reagent in a HyBEZ Hybridization System (ACD) for 30 minutes at 40°C.

IL6/IL6 receptor (IL6R) probes were added for 2-hour incubation at 40°C. The hybridization signals were amplified using the AMP 1 to 6 reagents provided. Slides were treated with AMP reagents at room temperature or 40°C for either 15 or 30 minutes, as detailed in the manufacturer's protocol. After the addition of AMP6, the slides were incubated in DAB for 10 minutes. Slides were washed and counterstained with 50% hematoxylin for 2 minutes and then washed again briefly in distilled water. Before mounting, the slides were dehydrated by incubating 2 minutes in 70% ethanol, twice 2 minutes in 95% ethanol, and 5 minutes in xylene. Coverslips were mounted onto slides using DPX mountant (Sigma-Aldrich). Slides were viewed under a bright-field panoramic digital slide scanner (3DHISTECH).

### Bioinformatic analyses

The Cancer Genome Atlas (TCGA) ovarian Affymetrix U133a 2.0 Array (18) was downloaded from UCSC Cancer Browser and the normalized gene expression dataset of clinically annotated primary, untreated ovarian tumor samples (*n* = 571) was extracted. The International Cancer Genome Consortium (ICGC) ovarian dataset (19) was extracted from the exp_seq.OV-AU.tsv.gz file from the ICGC Data Portal. Untreated, primary samples were used (*n* = 70). Only genes that achieved at least one read count in at least 10 samples were selected, producing 18,010 filtered genes in total and log_2_ counts per million (cpm) normalization was applied. Gene lists for HALLMARK_IL6_JAK_STAT3_SIGNALING, LU_TUMOR_ANGIOGENESIS_UP, KEGG_JAK_STAT_ SIGNALING_PATHWAY and REACTOME_PD-1_SIGNALING were downloaded from MSigDB genesets (http://www.gsea-msigdb.org/gsea/msigdb/genesets.jsp). Single sample gene-set enrichment analysis for these pathways, calculating a gene-set enrichment score per sample was performed using R package gene set variation analysis (GSVA; ref. 20). Heatmaps of GSVA scores with K-means row-clustering of the indicated pathways were constructed using R package ComplexHeatmap (21). ConsensusTME was applied on the normalized gene expression matrices to examine enrichment of tumor microenvironment cells in the TCGA and ICGC sample clusters (22).

### Quantification and statistical analysis

Stained tissue sections were scanned either with 3DHISTECH Panoramic 250 digital slide scanner or with Zeiss LSM 510 META confocal microscope. IHC staining quantification was carried using Definiens software (Definiens AG). Bioinformatic analyses were done using R language programming software. Qupath software was used for pSTAT3 nuclear tumor area analysis.

Statistical analyses were carried out using Graph pad prism software version 8. Data were tested to assess Gaussian distribution and two group data were analyzed using unpaired Student *t* test. Survival data were analyzed using Kaplan–Meier method using log-rank test. Multi-group statistical analyses were conducted using one-way ANOVA. The significance of all tests were defined as *, *P* < 0.05; **, *P* < 0.01; and ***, *P* < 0.001.

### Data and materials availability

All data associated with this study are provided with the submission of the main article or can be found in the Supplementary data file.

## Results

### Short-term cediranib treatment has immune modulating effects in mouse HGSOC models

Our aim was to understand the effects of cediranib on established tumor growth and model resistance to treatment. The orthotopic transplantable mouse models 30200 and 60577 share common genetic mutations (17, 23), replicate key aspects of the human TME, but vary in their average survival time and transcriptomes. Both models are syngeneic to FVB mice and primarily form omental tumors with time to humane endpoint of 6 to 8 weeks for 60577, and 4 to 6 months for 30200 (17). We previously found well-established omental tumors at 10 weeks in 30200 and as early as a week in 60577 after postintraperitoneal, tumor cell injection (17). Cediranib treatment commenced 10 weeks (30200) or 3 days (60577) after intraperitoneal injection and we measured effects on omental tumor growth and TME immunity after 4 to 5 weeks treatment. Treatment caused a significant reduction in omental weight (a surrogate for tumor burden; ref. 17) in both models (Fig. 1A, *P* < 0.05; Fig. 1B, *P* < 0.005). The average tumor burden decreased from 80 mg (30200) and 65 mg (60577) to 30 mg in both models. Flow cytometry analysis of tumors showed that cediranib caused significant changes in omental TME. There was a significant increase in CD3^+^ (*P* < 0.007), CD4^+^ (*P* < 0.01), CD8^+^ T (*P* < 0.02) cells and CD19^+^ B (*P* < 0.03) cells (Fig. 1A; Supplementary Fig. S1A) in the 30200 model. There was a concomitant decrease in F4/80^+^ macrophages (*P* < 0.005) but there were no differences in the expression of mannose receptor (MR) or MHC class II (MHCII) on myeloid cells (Supplementary Fig. S1A) suggesting there were no major changes in the phenotype of tumor-associated macrophages (TAM). In 60577 tumors there was also a significant increase in CD3^+^ (*P* < 0.008) and CD4^+^ T (*P* < 0.01) cells but no change in CD8^+^ T cells and CD19^+^ B cells after cediranib treatment (Fig. 1B; Supplementary Fig. S1B). In terms of myeloid cells, there was a significant increase in MR (*P* < 0.01) and MHCII (*P* < 0.005) but no difference in overall F4/80^+^ cells (Supplementary Fig. S1B).

Therefore, 4 to 5 weeks cediranib treatment decreased omental tumor burden and led to a 1.5- to two-fold increase in lymphocyte populations in both mouse models. We also observed a concomitant 1.5-fold decrease in TAMs in 30200 model and some changes in TAM phenotype in 60577 model.

To obtain insights into cediranib resistance, we continued the treatment to humane endpoint. In the 30200 model, continuation of cediranib treatment did not increase survival benefit or change the number and phenotype of immune infiltrate in omental tumors (Fig. 1C; Supplementary Fig. S1C). Moreover, postmortem examination revealed additional peritoneal metastases in some of the treated mice but none were found in controls (*P* < 0.04; Supplementary Fig. S1D).

In contrast, continued cediranib treatment significantly increased survival in 60577 mice with median survival increasing from 31 to 71 days (Fig. 1D). No differences were observed in MHCII but there was a significant increase in MR^+^ F4/80 (*P* < 0.0001) cells in treated tumors (Supplementary Fig. S1E). There was also a 1.5-fold decrease in CD3^+^ (*P* < 0.02), CD8^+^ (*P* < 0.03) and nearly six-fold decrease in CD19^+^ (*P* < 0.0006) cells but no change in CD4^+^ T cells (Fig. 1D; Supplementary Fig. S1E). No additional peritoneal metastases were recorded in the 60577-bearing mice.

Therefore, although the two models shared similar responses to short-term cediranib treatment, this was not sustained in 30200 model. An increase in peritoneal metastasis with no change in immune population at endpoint suggested acquired resistance to cediranib that may have been driven by malignant cells. Although there was a significant survival benefit with long-term cediranib treatment of 60577 tumors, the decline in T-lymphocyte populations suggested that eventual resistance involved inactivation of adaptive immune responses. We hypothesized that cediranib may induce different pathways of resistance in the two HGSOC models.

### Involvement of IL6 and STAT3 signaling in resistance to cediranib

**Figure 2.**
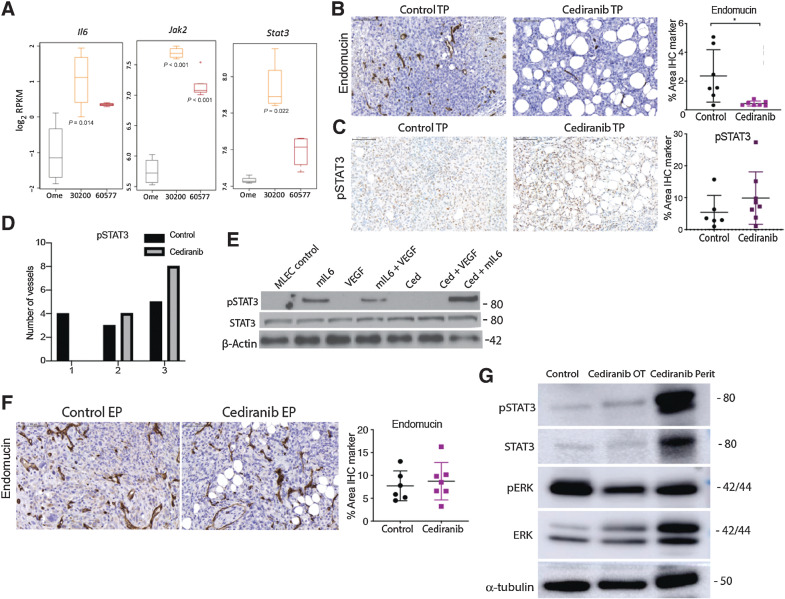
The IL6 pathway is involved in development of resistance to cediranib in 30200 model. **A,** Boxplots of log_2_ RPKM gene expression of indicated genes in omentum, 30200, and 60577 tumors (*n* = 4, 4, 5 respectively). **B,** IHC for endomucin and pSTAT3 in control and cediranib short-term treated 30200 tumors. **C,** IHC for pSTAT3 in control and cediranib short-term treated 30200 tumors. **D,** Intensity of pSTAT3 staining in control and cediranib short-term treated luminated vessels. **E,** Western blot analysis of pSTAT3, STAT3, and β-actin in MLEC treated with recombinant mIL6, VEGF, and cediranib. **F,** IHC for endomucin in control and cediranib-treated 30200 tumors at survival endpoint. **G,** Western blot analysis of pSTAT3, STAT3, pERK, ERK, and α-tubulin in 30200 control treated omental tumors, cediranib-treated omental, and peritoneal tumors collected at endpoint. Ome, omentum; ced, cediranib; EP, endpoint; perit, peritoneal; TP, short-term treated.

As activation of alternative signaling pathways such as IL6, IL8, and FGF upon inhibition of VEGFR signaling is proposed as a mechanism of acquired resistance to antiangiogenic therapies in some murine models and patients (24–26), we investigated these in our models. We focused on IL6 because we had previously shown that IL6 induced defective angiogenesis in HGSOC (8) and our work linked IL6 signaling with other angiogenic factors such as IL8 in an autocrine cytokine network in patients with HGSOC treated with anti-IL6 antibodies (7). Also, the 30200 and 60577 models had significant differences in levels of expression of the IL6 reactome pathway (17). Further interrogation of our RNA sequencing (RNA-seq) datasets revealed significantly higher levels of IL6, JAK2, and STAT3 mRNA in the 30200 tumors compared with 60577 tumors (Fig. 2A; Supplementary Fig. S2A). We stained for endomucin, as a measurement of blood vessel density, and pSTAT3 as the downstream target of IL6 in 30200 tumors from the 4- to 5-week treatment experiments. Endomucin-positive blood vessels were significantly decreased (Fig. 2B; *P* < 0.01) in treated tumors but there was no change in the overall pSTAT3 levels (Fig. 2C). However, we noticed some differences in pSTAT3 staining on the tumor vasculature, not in the percentage of positive pSTAT3 nuclei in luminated vasculature (Supplementary Fig. S2B), but in the proportion of luminated vessels with higher pSTAT3 staining intensity (*P* < 0.05) in the short-term cediranib-treated group compared with controls (Fig. 2D). This suggested a potential role of cediranib in potentiating IL6/STAT3 signaling in endothelial cells. Using recombinant IL6 and VEGF to mimic the paracrine mediators in tumors, we studied the direct effects of cediranib on MLECs. Western blot analysis on MLEC using various conditions of recombinant IL6, VEGF, and cediranib, revealed activation of pSTAT3 when mIL6 was present (Fig. 2E). We postulated that cediranib treatment in tumors with endogenous high IL6 levels could lead to enhanced pSTAT3 signaling on the endothelial cells. ELISA of supernatants revealed a concomitant increase in mIL6 level in the mIL6 plus cediranib group compared with mIL6 alone (Supplementary Figs. S2C).

To further understand cediranib-mediated IL6/pSTAT3 signaling on endothelial cells, we carried out a dose-response study with varying concentrations of recombinant mIL6 and found a dose-dependent increase in pSTAT3 levels when combined with cediranib (Supplementary Fig. S2D). These data suggested that cediranib-mediated effects on pSTAT3 signaling in endothelial cells arises in conditions with higher concentrations of IL6 and this could be involved in cediranib resistance in the 30200 model.

We extended these results using the rat aortic ring assay (8). Staining of vascular mural cells around the vessels revealed far fewer α-SMA-positive cells on the rat IL6 (rIL6) plus cediranib group as compared with VEGF and rIL6 alone (Supplementary Fig. S2E). As pericyte depletion was known to induce angiogenic growth and metastatic spread of disease, we speculated that STAT3 activation could be driving tumor angiogenesis and metastasis at survival endpoint in 30200 model as a result of cediranib-induced increased IL6 secretion and downstream activation of endothelial cells.

Unlike in short-term treated 30200 tumors, there was no change in vessel density or pSTAT3 expression with cediranib treatment in 30200 survival endpoint tumors (Fig. 2F). However, Western blot analysis revealed a slight increase in pSTAT3 in cediranib-treated omental tumors and a strong induction of pSTAT3 in cediranib-treated additional peritoneal tumors compared with the control omental tumors (Fig. 2G; Supplementary Fig. S2F). Conversely, there was a decrease in pERK levels in the cediranib-treated omental and peritoneal tumors compared with the control omental tumors (Fig. 2G). These data demonstrated sustained inhibition of the VEGF signaling in cediranib-treated tumors at survival endpoint but suggested potential escape and regrowth of peritoneal tumors via the IL6/STAT3 signaling. Activation of IL6 downstream signaling is a potential mode of resistance to cediranib in mouse models enriched for IL6 pathways.

### Combination of anti-IL6 and cediranib treatment increases mouse survival

**Figure 3.**
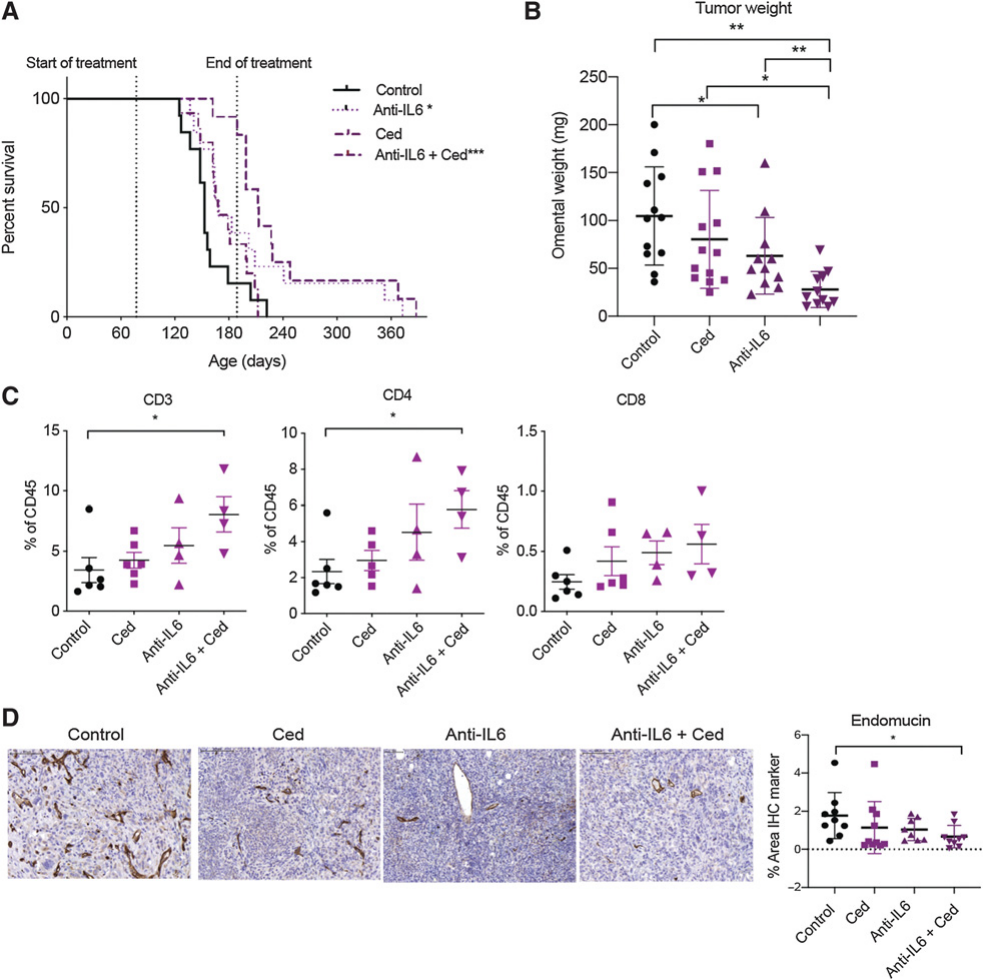
A combination of cediranib and anti-IL6 antibodies increases mouse survival in 30200 model. Mice injected with 30200 were treated with control, cediranib, anti-IL6, or combination of cediranib and anti-IL6. Vehicle control and cediranib was administrated by oral gavage 5 mg/kg five times a week, IgG control and anti-IL6 was given as i.p. 2 mg/kg twice a week. All treatment commenced at 10 weeks following cell injection and was carried on till endpoint or till maximum treatment duration as guided by home office license. **A,** Combined survival curve of 30200 model treated with cediranib, anti-IL6, or the combination (*n* = 12–13 per group). **B,** Quantification of omental weight across all groups from two experiments. **C,** Flow cytometric analysis of lymphoid infiltrate in all groups from one experiment. **D,** IHC staining for endomucin in all groups from two combined experiments. Ced, cediranib.

We next combined cediranib with a murine anti-IL6 blocking antibody that had immunomodulatory effects in the 30200 model (17). There was a significant increase in survival with anti-IL6 monotherapy (*P* < 0.03) and a more striking increase with combination group (*P* < 0.0002) as compared with controls. The median survival increased from 153 days in control group to 212 days in combination group with no formation of additional peritoneal metastasis (Fig. 3A). There was a 1.5-fold reduction in the omental tumor burden in anti-IL6 group (*P* < 0.03), and a four-fold reduction with combination treatment (*P* < 0.001), compared with control mice (Fig. 3B). There was a trend in decrease in F4/80 TAMs with combination therapy, however, significant decrease was only noted in the anti-IL6 (*P* < 0.03) group as compared with controls, as previously published (17) and an increase in MHCII expression in the combination group compared with anti-IL6 group (Supplementary Fig. S3A). In terms of lymphoid cells, although we did not observe any significant increases with either of the monotherapies, we noted significant increases in combination group compared with the controls. This included a —2- to 3 -fold significant increase in CD3^+^ (*P* < 0.03) and CD4^+^ T (*P* < 0.02) cells compared with controls (Fig. 3C) in endpoint tumors. There was also a trend to an increase of CD8^+^ T cell in the combination arm (*P* < 0.06) as compared with control but no differences observed in CD19^+^ cells across the different groups (Fig. 3C; Supplementary Fig. S3A). In conclusion, by combining anti-IL6 with cediranib, we were able to significantly increase survival and T-lymphocyte populations in the TME. Vessel density was significantly reduced at endpoint in the combination arm (*P* < 0.04) compared with control and monotherapies (Fig. 3D) as were a two-fold decrease in malignant cell marker cytokeratin 8 (CK8; *P* < 0.001), but no change in proliferating Ki67-positive cells (Supplementary Figs. S3B). There were no overall differences in pSTAT3 staining between the groups at endpoint (Supplementary Fig. S3C), however tumors in the cediranib (*P* < 0.02), anti-IL6 (*P* < 0.02), and combination group (*P* < 0.0002) displayed less pSTAT3 expression in malignant cell area compared with stroma (Supplementary Fig. S3C) which was greatest with the combination therapy (Supplementary Fig. S3D).

Therefore, resistance to cediranib in the 30200 model could be partially overcome by inhibiting IL6 signaling; combination treatment increased survival, reduced tumor burden, and angiogenesis.

### Cediranib and anti-IL6 antibody treatment increases survival in a second murine HGSOC model

**Figure 4.**
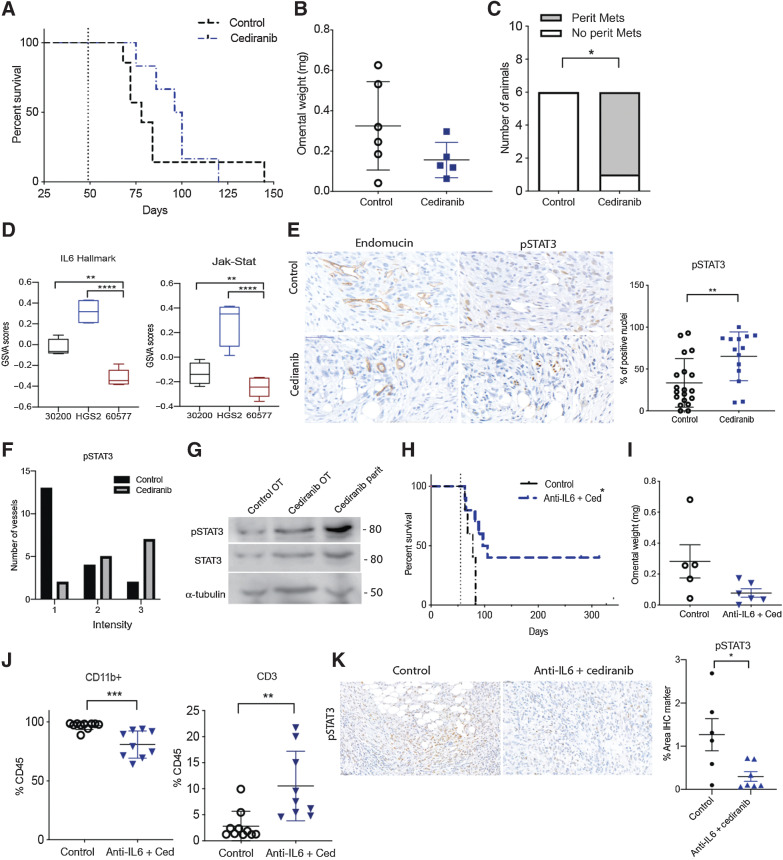
A combination of cediranib and anti-IL6 also increase mouse survival in HGS2 model of HGSOC. Mice injected with HGS2 cells were treated with vehicle control or cediranib oral gavage 5 mg/kg five times a week starting 8 weeks after cell injection until endpoint. **A,** Survival curve of cediranib-treated HGS2 model (*n* = 7 per group). **B,** Quantification of tumor weight in omentum. **C,** Analysis of peritoneal metastasis in cediranib-treated HGS2 tumors at endpoint. **D,** Boxplot of GSVA enrichment scores for 30200, HGS2, and 60577 (*n* = 4, 4, 5 respectively). **E,** pSTAT3 staining on rumanted vasculature in control and cediranib survival tumors. **F,** Intensity of pSTAT3 staining in control and cediranib endpoint treated vessels. **G,** Western blot analysis of pSTAT3, STAT3 α-tubulin in HGS2 control treated omental tumors, cediranib-treated omental, and peritoneal tumors collected at endpoint. Mice injected with HGS2 cell were treated with control or combination of cediranib plus anti-IL6. Vehicle control and cediranib were administrated by oral gavage 5 mg/kg five times a week, IgG control and anti-IL6 were given as i.p. 2 mg/kg twice a week. All treatment commenced at 7.8 weeks following cell injection and was carried on till endpoint. **H,** Survival curve of HGS2 model treated with control or combination (*n* = 7 per group). **I,** Analysis of tumor weight in control and combination treated HGS2 model at endpoint. **J,** Flowcytometric analysis of lymphoid and myeloid population in combination treated HGS2 tumors. **K,** IHC staining for pSTAT3 in control and the combination treated group. Perit, peritoneal; Mets, metastasis; OT, omental tumors; Ced, cediranib.

We next investigated the combination of cediranib and anti-IL6 in another orthotopic murine HGSOC, HGS2 (17). Cediranib treatment of established peritoneal tumors commenced 7 weeks after intraperitoneal injection of tumor cells and continued until humane endpoint. There was no significant effect on mouse survival (Fig. 4A) nor omental tumor burden (Fig. 4B) and, as with 30200, we found additional peritoneal metastases in the cediranib-treated group that were not found in control mice (*P* < 0.02; Fig. 4C).

HGS2 tumors had highest levels of IL6 pathway gene expression compared with 30200 or 60577 models (Fig. 4D). Therefore, we investigated pSTAT3 staining in luminated vasculature and found a significant increase in both the percentage of positive pSTAT3 nuclei (*P* < 0.004) and intensity of pSTAT3 staining (*P* < 0.05) in cediranib-treated vessels compared with controls (Fig. 4E and F). Western blot analysis revealed a slight increase in pSTAT3 signaling in the cediranib-treated omental tumors and a much higher increase of pSTAT3 in the cediranib-treated additional peritoneal tumors, similar to the 30200 tumors (Fig. 4G).

These data suggested that HGS2 tumors may also acquire resistance to cediranib-therapy via the activation of IL6 signaling. We previously showed no beneficial effect on survival with anti-IL6 antibody therapy in the HGS2 model (17). However, combination of cediranib and anti-IL6 antibody treatment of established tumors significantly increased mouse survival with some mice surviving 320+ days after start of the experiment (Fig. 4H). In the mice that eventually developed tumors, there was a trend towards reduced omental tumor weight and no additional peritoneal tumors were seen (Fig. 4I). Flow cytometric analysis of the TME revealed a four-fold increase in CD3^+^ T cells (*P* < 0.003) and a significant decrease in CD11b^+^ myeloid cells (*P* < 0.0005) in combination arm compared with controls (Fig. 4J). IHC staining for pSTAT3 also revealed a significant reduction in the combination of anti-IL6 and cediranib treated omental tumors compared with controls (*P* < 0.02; Fig. 4K). Taken together, the results suggest that anti-IL6 treatment may abrogate cediranib resistance and that we may be able to predict treatments that successfully combine with cediranib by interrogating the intrinsic gene expression pathways of advanced untreated tumors, at least in these mouse models.

We predicted that in tumors with low intrinsic levels of IL6 signaling, such as seen in 60577, cediranib would be able to inhibit both VEGF and IL6 signaling pathways. In support of this, we found a significant decrease in angiogenesis in long term cediranib-treated omental 60577 tumors as measured by endomucin staining (*P* < 0.0001) and a significant decrease in STAT3 activation (*P* < 0.0009; Supplementary Figs. 4A).

There were significant differences in immune checkpoint pathways between 60577 and 30200 models in our RNA-seq data especially in the PD-1 reactome pathway (Supplementary Fig. 4B). Flow cytometric analysis of cediranib-treated 60577 tumors at endpoint revealed a significant increase in PD-1 levels on CD3^+^ (*P* < 0.01) and CD19^+^ (*P* < 0.04) cells and significant increase in PD-L1^+^MR^+^ macrophages (*P* < 0.0001; Supplementary Fig. 4C). This suggested that upregulation of PD-1 signaling might be involved in resistance to cediranib in 60577 model.

### Combining anti–PD-1 antibody with cediranib increases survival of 60577-bearing mice

**Figure 5.**
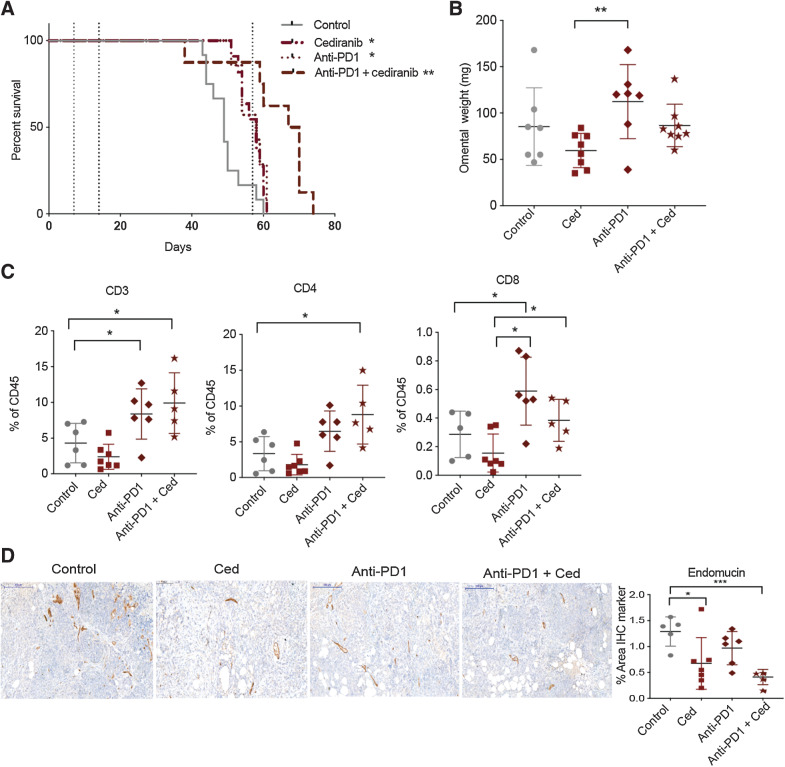
Combination of anti–PD-1 and cediranib increases survival in 60577 model. Mice injected with 60577 cells were treated with control, cediranib, anti–PD-1, or combination of cediranib plus anti–PD-1. Vehicle control and cediranib were administrated by oral gavage 5 mg/kg five times a week, IgG control and anti–PD-1 were given i.p. 2 mg/kg twice a week. Cediranib treatment commenced a week after cell injection and was continued till endpoint, anti–PD-1 treatment started at 2 weeks following cell injection and was given for a period of 6 weeks (indicated by dotted lines). **A,** Survival curve of 60577 model treated with cediranib, anti–PD-1, or the combination (*n* = 7 per group). **B,** Quantification of omental weight at endpoint. **C,** Flow cytometric analysis of lymphoid infiltrate in all treated groups. **D,** IHC staining for endomucin in all groups. Ced, cediranib.

Anti–PD-1 antibody treatment of established 60577 tumors started a week after cediranib treatment began and continued for 6 weeks at which point the anti–PD-1 treatment was stopped due to signs of toxicity. Cediranib treatment continued to humane endpoint. Cediranib and anti–PD-1 antibodies monotherapies significantly increased survival in 60577 (median survival 58 days compared with 49 in control) model but combination of the two agents had a greater effect (median survival 68 days; *P* < 0.001; Fig. 5A). There was an almost two-fold significant increase in omental weight, possibly due to infiltration of immune cells, in anti–PD-1 group compared to cediranib group (*P* < 0.005) but no significant changes with the combination treatment at endpoint (Fig. 5B). The increases in survival in treatment arms were accompanied with changes in tumor immune infiltrate. There was a significant increase in CD3^+^ T cells in anti–PD-1 (*P* < 0.05) and combination arm (*P* < 0.02) as compared with the control group (Fig. 5C). This significant increase correlated with a significant increase in CD4^+^ T cells in combination group (*P* < 0.02) (Fig. 5C; Supplementary Fig. S5A). Notably, there was a significant increase in CD8^+^ cells in combination group (*P* < 0.02) as compared with cediranib alone, suggesting that anti–PD-1 treatment can rescue the dampening of CD8^+^ T-cell numbers observed with cediranib-therapy alone. In addition, there was a 1.5- to two-fold significant decrease in vessel density in cediranib arm (*P* < 0.03) as previously observed in Supplementary Fig. 4A as well as a more marked three-fold decrease in combination group (*P* < 0.0001; Fig. 5D). There were no changes in Ki67-positive proliferating cells and there was a decreasing trend in CK8^+^ tumor cells in combination arm (*P* < 0.07) compared with control (Supplementary Fig. S5B). In summary, a combination of cediranib and anti–PD-1 enhanced the survival benefits and overcome the protumoral response mediated by cediranib treatment in 60577 model.

### Analysis of IL6, PD-1, and angiogenesis pathways in human HGSOC tumors show translational significance of mouse model data

**Figure 6.**
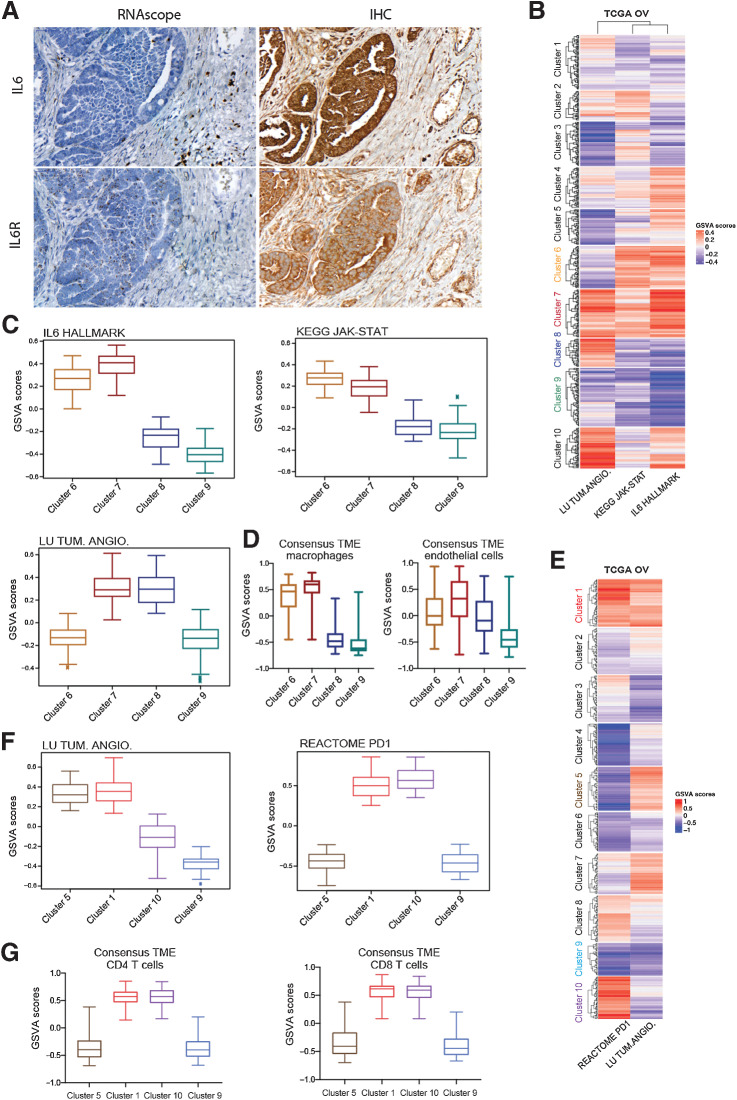
Clustering of TCGA dataset based on angiogenesis, IL6, and PD-1 pathway expression. **A,** RNAscope and IHC for IL6 and IL6R in HGSOC patient biopsies. **B,** Heatmap illustrates GSVA enrichment scores for Lu tumor angiogenesis up, Kegg JAK-STAT signaling pathway, and Hallmark IL6-JAK-STAT signaling from MSigDB calculated for each sample of the TCGA ovarian dataset (*n* = 571). **C,** Boxplots illustrate GSVA scores for Lu tumor angiogenesis up, Kegg JAK-STAT signaling pathway, and Hallmark IL6-JAK-STAT across clusters 6, 7, 8, and 9 representing four major patterns of pathways’ expression (*n* = 57, 65, 38, 79 respectively). **D,** ConsensusTME applied on sample clusters identified in (**B**). Boxplots illustrate GSVA scores for macrophages and endothelial cells across the four clusters of interest. **E,** Heatmap illustrates GSVA enrichment scores for Lu tumor angiogenesis up and Reactome PD-1 signaling calculated for each sample of TCGA ovarian dataset. **F,** Boxplots illustrate GSVA scores for Lu tumor angiogenesis up and Reactome PD-1 signaling across clusters 5, 1, 10, and 9 representing the four major expression patterns of these pathways (*n* = 46, 65, 76, 43 respectively). **G,** ConsensusTME was applied on the sample clusters identified in (**E**). Boxplots illustrate GSVA scores for CD4 and CD8 T cells across four clusters of interest.

We next wanted to investigate the signaling pathways behind these mechanisms in human HGSOC and to see if there is a similar variation between patients. There are a number of reports relating to the PD-1 pathway and its significance on immune status in HGSOC (27–29). PD-1 and PD-L1 protein is found in HGSOC biopsies, with PD-L1 primarily found on macrophages and some malignant cells and PD-1 generally found on lymphocytes (27, 29). With limited data on the cellular location of IL6 and its gp80 receptor in HGSOC, we conducted RNAscope and IHC analysis on HGSOC biopsies. IL6 mRNA was predominantly stromal and IL6R mRNA expression mainly in malignant cell areas. IL6 and IL6R protein were found predominantly in malignant cell areas with some stromal staining (Fig. 6A). We also performed IHC multiplex staining for IL6, PAX8 (HGSOC tumor marker), and CD3 on HGSOC patient biopsies and observed IL6 brown staining colocalized in areas with purple tumor cells and some blue tumor-infiltrating lymphocytes (Supplementary Fig. 6A).

If the findings in our mouse models were relevant to patients, we should be able to find subsets of HGSOC tumors that resembled the variation in transcriptional pathways found in mouse tumors. We interrogated two publicly available transcriptional datasets from primary HGSOC biopsies studying levels of the IL6, JAK-STAT, and PD-1 pathways and assessed their relationship to levels of angiogenesis and immune cell pathways. We conducted clustering analysis of the TCGA (18) ovarian cancer dataset based on GSVA enrichment scores (20) of IL6, JAK-STAT, and tumor angiogenesis pathways and identified 10 clusters with distinct expression patterns (Fig. 6B; Supplementary Table S1A). Of particular interest were clusters 6 to 9: cluster 6 presented elevated IL6/JAK-STAT with low angiogenesis; cluster 7 high IL6/JAK-STAT and high angiogenesis; cluster 8 low IL6/JAK-STAT and elevated angiogenesis; cluster 7 low IL6/JAK-STAT and low angiogenesis (Fig. 6C). Among these subgroups of patients, clusters 6 and 7 closely resembled IL6 and JAK-STAT signaling pattern found in 30200 and HGS2 mouse models whilst clusters 8 and 9 resembled the 60577 transcriptome. We next asked whether high IL6-, JAK-STAT–expressing human biopsies shared additional TME features with the respective mouse models using ConsensusTME (22). This revealed macrophages and endothelial cells to be enriched in high IL6 clusters compared with low IL6 clusters (Fig. 6D; Supplementary Table. S1B). This correlated with our previous multi-scale study on the mouse models which revealed 30200 and HGS2 tumors with highest number of macrophages compared to low IL6-expressing model 60577 (17). Furthermore, higher enrichment in endothelial cells in high IL6 clusters again associated with the signature found in high IL6-expressing murine models.

We repeated the same analysis on the ICGC (19) ovarian cancer dataset. We could again identify patient clusters with similar patterns of high and low IL6, JAK-STAT, and angiogenesis signatures (Supplementary Fig. S6A and B; Supplementary Table S2A). The pattern of TME macrophage and endothelial characteristics also correlated with high IL6 and JAK-STAT signature as seen in TCGA dataset (Supplementary Fig. S6C; Supplementary Table. S2B).

We then subgrouped patients with HGSOC based on their PD-1 and angiogenesis signatures. Unsupervised clustering was applied to the TCGA dataset using the GSVA scores of the PD-1 and tumor angiogenesis pathways (Fig. 6E; Supplementary Table. S1C). Of particular interest to us were clusters 1,10, 5, and 9, first two displaying high levels of PD-1 with high or low angiogenesis and latter two exhibiting low PD-1 expression with high or low angiogenesis (Fig. 6E and F). ConsensusTME analysis showed that high PD-1 clusters 1 and 10 were also enriched for the CD4 and CD8 signatures unlike the other clusters with low PD-1 levels (Fig. 6G; Supplementary Table S1D). This directly translates into our mouse models where we found that one of the defining characteristics of the 60577 tumors is higher infiltration of T lymphocytes compared with 30200 and HGS2 (17).

We further validated these findings in ICGC dataset where we found similar clusters of PD-1 and angiogenesis signaling patterns (Supplementary Fig. S6D and S6E; Supplementary Table S2C). Again, clusters with high enrichment of the PD-1 reactome pathway were also enhanced for T-lymphocyte signaling with increase in CD4 and CD8 expression (Supplementary Fig. S6F; Supplementary Table S2D).

In conclusion, analysis of ICGC and TCGA transcriptional datasets revealed strong correlations between high levels of angiogenesis signatures, the IL6 and PD1 pathways and immune cell signatures, indicating that our findings in mouse models could be translated to subsets of patients with HGSOC.

## Discussion

In preclinical experiments using established peritoneal tumors from three different mouse models of HGSOC, we have identified two pathways of cediranib-mediated resistance and confirmed the significance of these data with combination therapies by analysis of transcriptomic databases from patient samples. Previous studies with antiangiogenic agents have reported immunostimulatory effects with increase in immune infiltration as a result of transient physical changes on tumor vasculature due to vessel normalization and alleviation of hypoxia (30). This correlated with our short-term cediranib studies that showed an increase in T-lymphocyte populations and further supported previous findings on the immunosuppressive roles of VEGF pathway in tumors (31, 32). Conversely, the opposite is observed where prolonged treatment with antiangiogenic agents lead to vessel damaging effects enhancing metastasis and reduced penetration of the tumor by immune cells (33). In our models with higher levels of intrinsic IL6 signaling pathways (30200 and HGS2), we observed this deleterious effect with increase in metastasis and reverting of tumor suppressive immune cell phenotype following cediranib-treatment at endpoint.

Sustained vessel normalization with the blockade of angiopoeitin-2 (ANG2) along with VEGF inhibition has been reported as a more effective strategy in increasing pericyte coverage and improving functionality of tumor vasculature (34). In concordance to this, we have previously shown IL6 can drive destabilized vasculature with loss of pericytes via activation of the ANG2 signaling and that anti-IL6 therapy can normalize this effect in xenograft models of ovarian cancer (8). We therefore believe at least in 30200 and HGS2, a robust effect on reprogramming the tumor vasculature and immune surveillance following cediranib-therapy was achieved with a combination of anti-IL6 agents.

The immune-mediated effects not only varied between the different models but also within same model after short versus sustained treatment with cediranib. Anti-VEGF therapy-induced inhibition of adaptive immune response as a result of enriched PD-1 signaling on immune cells was seen in 60577 model. The dampening of T-cell response observed with long term cediranib treatment that facilitated anti–PD-1 combination in 60577 model, was a similar mechanism that dictated successful combination of PARP inhibitors with anti–PD-1 therapy in ovarian cancer patients (35).

The potential cediranib combinations uncovered with anti-IL6 and anti–PD-1 antibodies are therapies that are approved and routinely used in clinic for various diseases. Anti-IL6 therapies are currently used to treat conditions like uveitis, neuromyelitis optica, and most recently, COVID-19 pneumonia (36). Phase II studies of anti-IL6 antibody siltuximab, in metastatic castration resistant prostate cancer and platinum resistance ovarian cancer have shown limited, but some biological effect in the latter trial, indicating potential for blocking this pathway in cancers (7, 37). More recent studies using gene expression analysis of patients with prechemotherapy treated gastric cancer identified an IL6 stromal signature predictive of poor response to chemotherapy (38). This study again highlighted the importance of selecting subgroups of patients that would benefit from these biological interventions.

Gene expression analysis of HGSOC patient clusters enriched for angiogenesis, IL6, JAK-STAT, and PD-1 pathways that shared similarities in characteristics with the mouse models of interest, in terms of macrophage, endothelial and T-cell phenotypes provided further validation of the models and drug combinations.

This study also heightens the need for identifying specific clusters of patients that share intrinsic molecular signatures, thereby allowing for better stratification and design of combinations following antiangiogenic treatment in HGSOC.

## Supplementary Material

Supplementary Figure

Supplementary Table

Supplementary Table
