# Supplementary material for: Immune Mechanisms of Resistance to Cediranib in Ovarian Cancer
Source: Mol Cancer Ther. 2022 Mar 21;21(6):1030–43. doi: 10.1158/1535-7163.MCT-21-0689 (PMC9167758; doi:10.1158/1535-7163.MCT-21-0689)
Supplement: Supplementary Figure [file mct-21-0689_supplementary_figures_1-6_suppsf1-6.docx]

**Supplementary Data**

**Immune mechanisms of resistance to cediranib in ovarian cancer**

Ganga Gopinathan, Chiara Berlato, Anissa Lakhani, Ludmila Szabova, Colin Pegrum, Ana-Rita Pedrosa, Florian Laforets, Eleni Maniati, Frances Balkwill

*Corresponding author. Email: f.balkwill@qmul.ac.uk

**This file includes:**
Supplementary Figs. S1. **Short-term treatment and survival effects of cediranib on immune microenvironment in two mouse models of HGSOC**
Supplementary Figs. S2. **IL-6 pathway mediated resistance to cediranib therapy.**
Supplementary Figs. S3. **Effects of Anti-IL-6 and cediranib combination on 30200 TME**

Supplementary Figs. S4. **A different mechanism of resistance to cediranib in 60577 HGSOC model**

Supplementary Figs. S5. Effects of Anti-PD1 and cediranib combination on 60577 TME
Supplementary Figs. S6. Clustering of ICGC dataset based on Angiogenesis, IL-6 and PD1 pathway expression

**Other Supplementary data for this manuscript includes the following:**

Supplementary Table S1 and S2 (Microsoft Excel format). Individual data points.


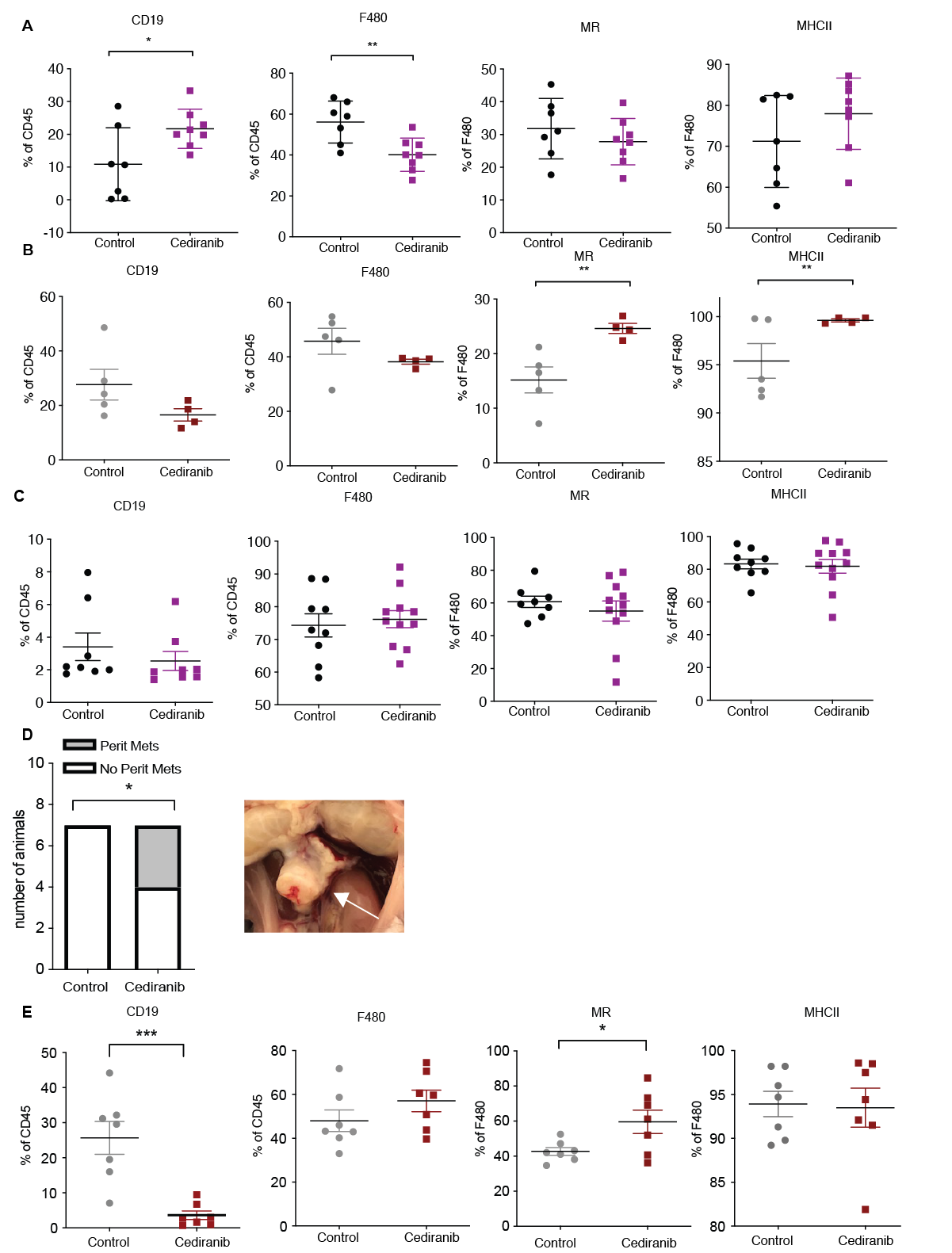


**Supplementary Figs. S1: Short-term treatment and survival effects of cediranib on immune microenvironment in two mouse models of HGSOC.**

10x10^^6^ cells were injected i.p. into immunocompetent syngeneic FVB mice. Mice injected with 30200 or 60577 were treated with vehicle control or cediranib oral gavage 5 mg/kg five times a week starting at 10 weeks (30200) or 3 days (60577) after cell injection for a period of 4-5 weeks (short-term treatment) or until end point (survival). **A.** Flowcytometric analysis of CD19 and myeloid infiltrate markers F4/80, MHCII and MR in 30200 omental short-term treated tumors. **B.** Flowcytometric analysis of CD19 and myeloid infiltrate markers F4/80, MHCII and MR in 60577 omental short-term treated tumors. **C.** Flowcytometric analysis of CD19 and myeloid infiltrate markers F4/80, MHCII and MR in 30200 omental tumors at end point. D. Additional peritoneal metastasis found in 30200 cediranib treated mice at end point. E. Flowcytometric analysis of CD19 and myeloid infiltrate markers F4/80, MHCII and MR in 60577 omental tumors at end point. ( * p < 0.05, ** p < 0.01, *** p < 0.001). Each dot represents a tumor from an individual mouse.


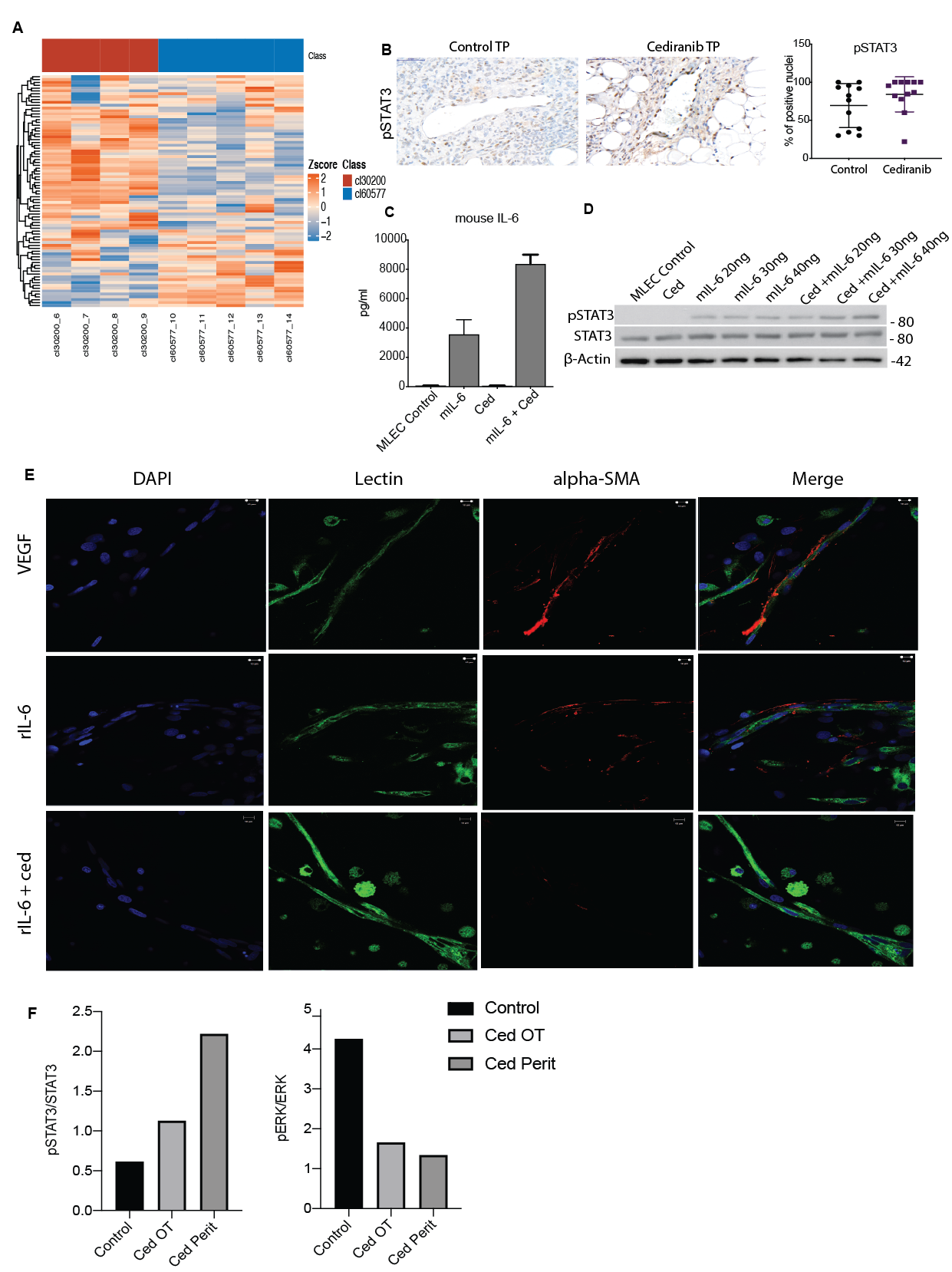


**Supplementary Figs. S2: IL-6 pathway mediated resistance to cediranib therapy.**

**A.**Heatmap of row z-scores of log_2_RPKM gene expression of Hallmark IL6-JAK-STAT signaling expression MSigDB gene-set. **B**. pSTAT3 staining on luminated vasculature in control and cediranib short-term treated tumors.

**2 x 10^5^ MLEC cells were plated and treated with recombinant IL-6 , VEGF and cediranib. C. ELISA quantification of mIL-6 in conditioned medium from MLEC treated with recombinant mIL-6 (30ng/ml), VEGF (30ng/ml) and cediranib (100nM) for 24 hours. D.** Western blot analysis of pSTAT3, STAT3 and β-actin from MLEC protein lysates treated with varying concentrations of recombinant mIL-6 (20, 30 and 40ng/ml) plus cediranib (100nM) for 24 hours. **E.** Rat aortic ring vessels treated with recombinant rIL-6 (10ng/ml), VEGF (10ng/ml) and cediranib (10nM) stained for endothelial cells using BS1 lectin (green), α-SMA (red) and DAPI (blue). **F.** Densitometry analysis for western blot shown in Figure 2G.


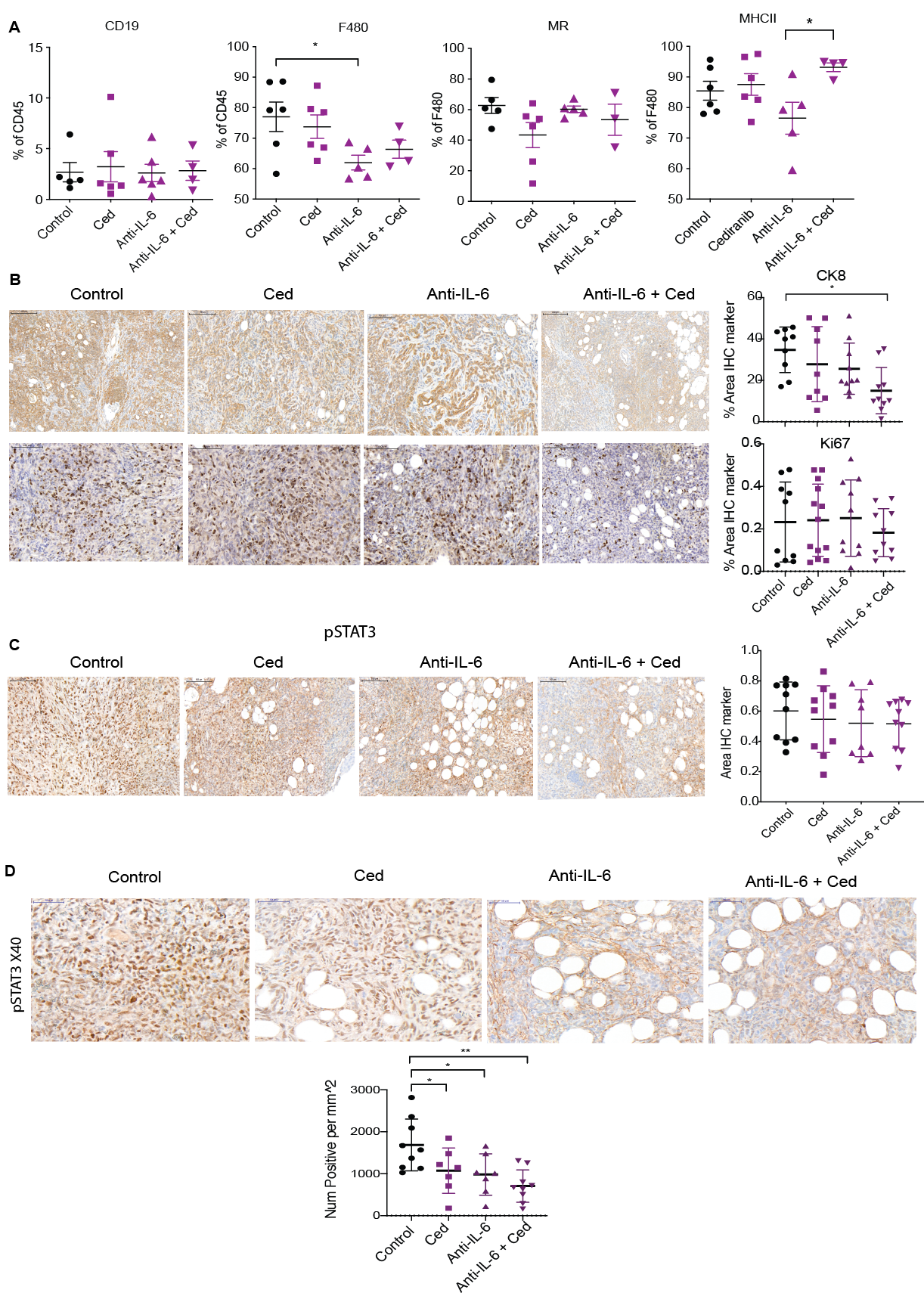


**Supplementary Figs. S3: Effects of Anti-IL-6 and cediranib combination on 30200 TME**

10x10^^6^ cells were injected i.p. into immunocompetent syngeneic FVB mice. Mice injected with 30200 were treated with control (vehicle control and IgG control), cediranib, anti-IL-6 or combination of cediranib and anti-IL-6. Vehicle control and cediranib was administrated by oral gavage 5 mg/kg five times a week, IgG control and anti-IL-6 was given as i.p. 2mg/kg twice a week. All treatment commenced at 10 weeks following cell injection and was carried on till end point or till maximum treatment duration as guided by home office license. **A.**  Flowcytometric analysis of CD19 and myeloid infiltrate markers F4/80, MHCII and MR in 30200 omental tumors at end point. **B.** IHC staining for CK8 and Ki67 in all groups. **C.** IHC staining for pSTAT3 in all groups. Student’s t test value is depicted on the scatter plots ( * p < 0.05, ** p < 0.01, *** p < 0.001). Each dot represents a tumor from an individual mouse. IHC analysis carried out by quantification of the percentage of positive area using the Definiens Tissue studio.


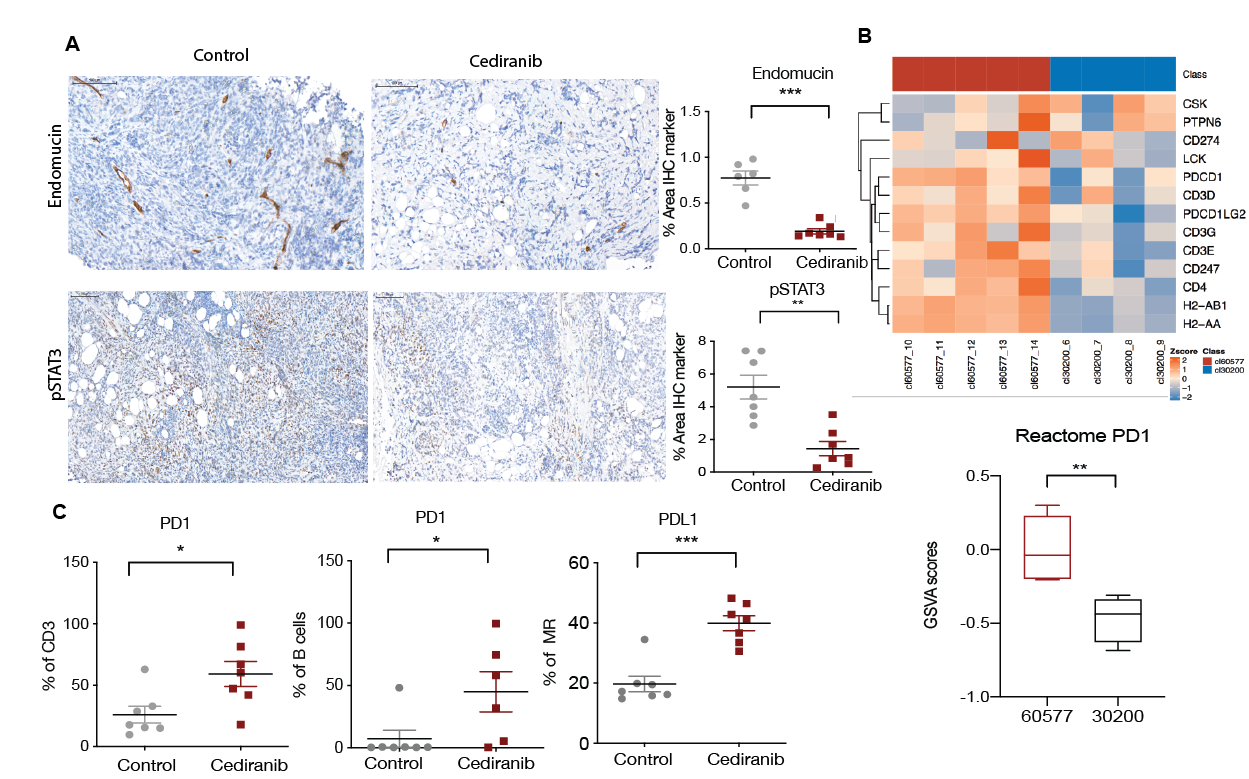


**Supplementary Figs. S4: A different mechanism of resistance to cediranib in 60577 HGSOC model**

**
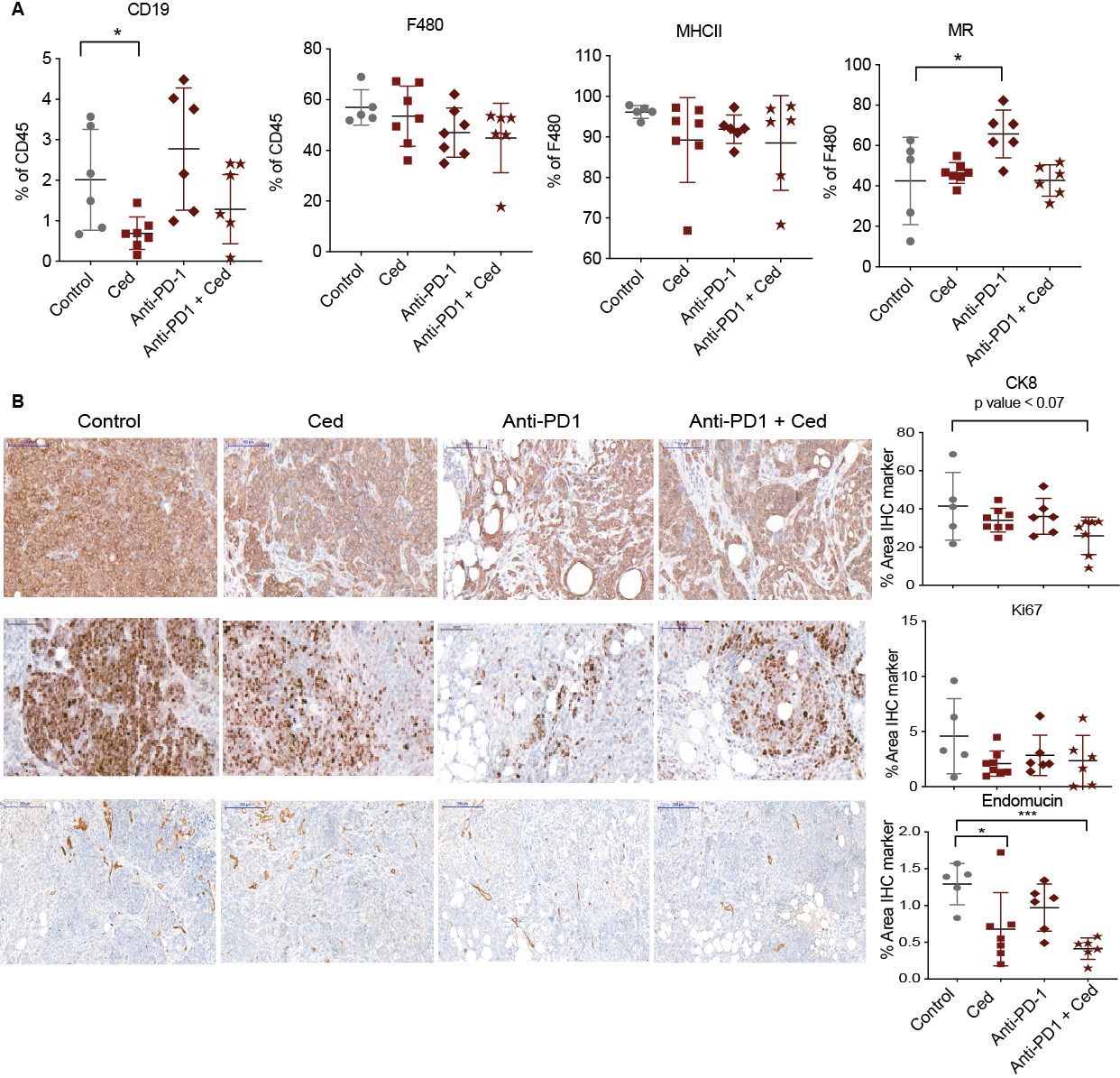
A.** IHC for endomucin and pSTAT3 in control and cediranib-treated 60577 tumors at survival end point. **B**, Heatmap of row z-scores of Reactome PD1 pathway gene expression and boxplot of GSVA enrichment scores of the same pathway for 30200 and 60577 (GSE71349 n=4,5 respectively) **C.** Flowcytometric analysis of PD1 signaling in 60577 omental immune infiltrate at end point.

**Supplementary Figs. S5: Effects of Anti-PD1 and cediranib combination on 60577 TME**

10x10^^6^ cells were injected i.p. into immunocompetent syngeneic FVB mice. Mice injected with 60577 cells were treated with control (vehicle control and IgG control), cediranib, anti-PD1 or combination of cediranib plus anti-PD1. Vehicle control and cediranib were administrated by oral gavage 5 mg/kg five times a week, IgG control and anti-PD1 were given i.p. 2mg/kg twice a week. Cediranib treatment commenced a week after cell injection and was continued till end point, anti-PD-1 treatment started at 2 weeks following cell injection and was given for a period of 6 weeks. **A.** Flowcytometric analysis of CD19 and myeloid infiltrate markers F4/80, MHCII and MR in 60577 omental tumors at end point. **B.** IHC staining for CK8 and Ki67 in all groups. Student’s t test value is depicted on the scatter plots ( * p < 0.05, ** p < 0.01, *** p < 0.001).). Each dot represents a tumor from an individual mouse. IHC analysis carried out by quantification of the percentage of positive area using the Definiens Tissue studio.


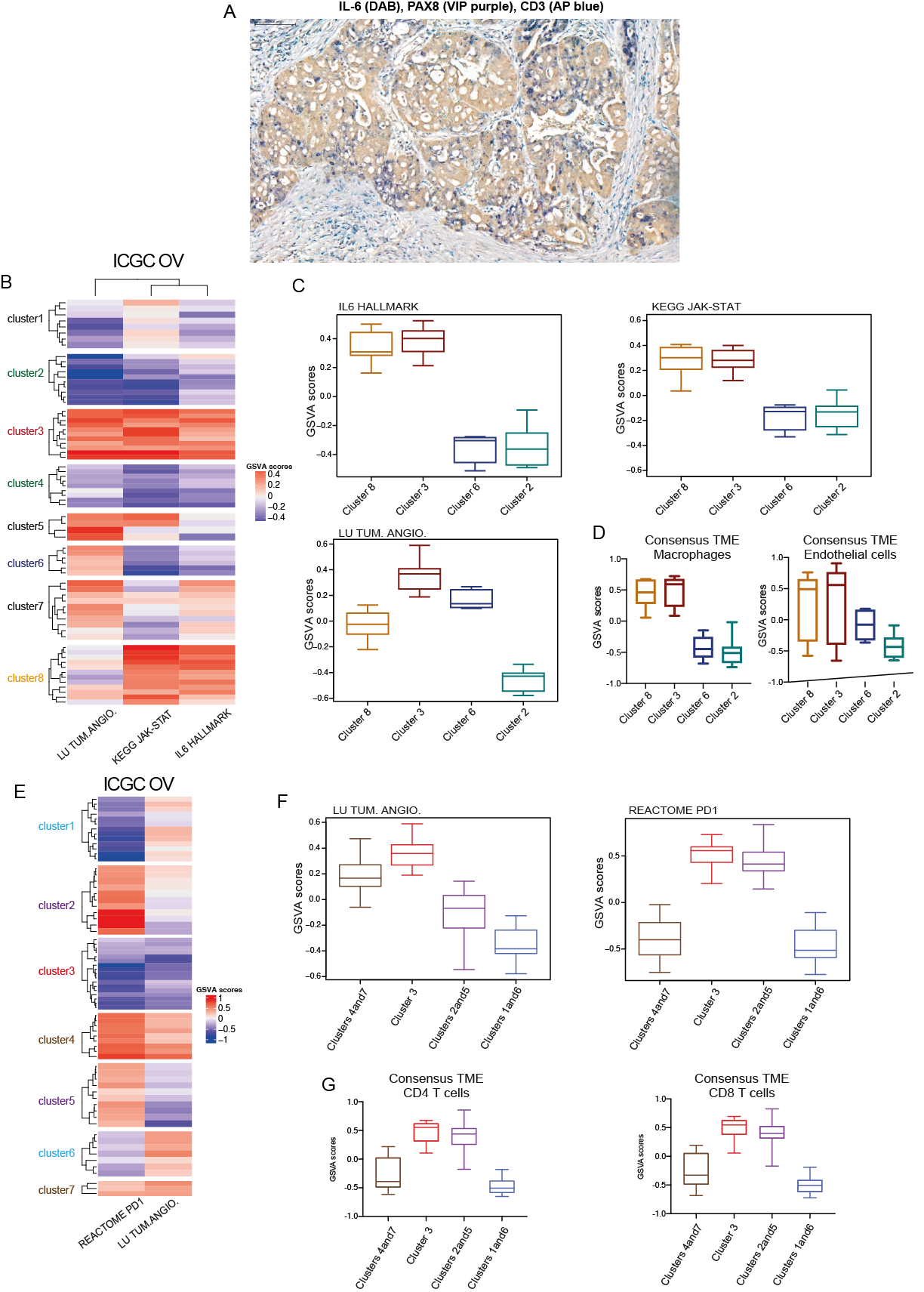


**Supplementary Figs. S6: Clustering of ICGC dataset based on Angiogenesis, IL-6 and PD1 pathway expression.**

**A.** Human HGSOC biopsies stained for IL-6 (DAB-brown), PAX8 (VIP-purple) and CD3 ( AP blue). **B** Heatmap illustrates GSVA enrichment scores for Lu tumor angiogenesis up), Kegg JAK-STAT signaling pathway and Hallmark IL6-JAK-STAT signaling calculated for each sample of the ICGC ovarian dataset (n = 70, primary samples, untreated). K-means row clustering was applied to identify groups of samples with distinct expression patterns of the indicated pathways of interest. **C**. Boxplots illustrate GSVA scores for LU Tum Lu tumor angiogenesis up, Kegg JAK-STAT signaling pathway and Hallmark IL6-JAK-STAT signaling across clusters 8, 3, 6 and 2 representing the four major patterns of pathways’ expression (n = 12, 11, 6 and 10 respectively). **D**. ConsensusTME was applied on sample clusters identified in A. Boxplots illustrate GSVA scores for macrophages and endothelial cells across the four clusters of interest. **E**. Heatmap illustrates GSVA enrichment scores for Lu tumor angiogenesis up and Reactome PD1 signaling calculated for each sample of the ICGC ovarian dataset. K-means row clustering was applied to the samples (rows) of the heatmap. **F**. Boxplots illustrate GSVA scores for Lu tumor angiogenesis up and Reactome PD1 signaling across clusters 4 and 7 (n = 12), 3 (n = 17), 2 and 5 (n = 21), 1 and 6 (n = 20) representing the four major expression patterns of these pathways, having merged clusters with similar expression profiles. **G**. ConsensusTME was applied on the sample clusters identified in D. Boxplots illustrate GSVA scores for CD4 and CD8 T cells across the four merged clusters of interest.
